# Effect of leisure time physical activity on severe knee or hip osteoarthritis leading to total joint replacement: a population-based prospective cohort study

**DOI:** 10.1186/1471-2474-13-73

**Published:** 2012-05-17

**Authors:** Eva Ageberg, Gunnar Engström, Maria Gerhardsson de Verdier, Jan Rollof, Ewa M Roos, L Stefan Lohmander

**Affiliations:** 1 Department of Orthopedics, Clinical Sciences Lund, Lund University, Lund, Sweden; 2 Department of Health Sciences, Lund University, Lund, Sweden; 3 AstraZeneca R&D, Lund, Molndal, Sweden; 4 Epidemiology Research Group, Clinical Sciences, Malmö, Lund University, Lund, Sweden; 5 Institute of Sports Science and Clinical Biomechanics, University of Southern Denmark, Odense, Denmark; 6 Department of Orthopedics and Traumatology, University of Southern Denmark, Odense, Denmark; 7 Department of Health Sciences, Lund University, PO Box 157, SE-221 00, Lund, Sweden

**Keywords:** Osteoarthritis, Arthroplasty, Exercise, Workload, Risk factors

## Abstract

**Background:**

Studies on leisure time physical activity as risk factor or protective factor for knee or hip osteoarthritis (OA) show divergent results. Longitudinal prospective studies are needed to clarify the association of physical activity with future OA. The aim was to explore in a prospective population-based cohort study the influence of leisure time physical activity on severe knee or hip OA, defined as knee or hip replacement due to OA.

**Methods:**

Leisure time physical activity was reported by 28320 participants (mean age 58 years (SD 7.6), 60% women) at baseline. An overall leisure time physical activity score, taking both duration and intensity of physical activities into account, was created. The most commonly reported activities were also used for analysis. The incidence of knee or hip replacement due to OA over 11 years was monitored by linkage with the Swedish hospital discharge register. Cox’s proportional hazards model (crude and adjusted for potential confounding factors) was used to assess the incidence of total joint replacement, or osteotomy (knee), in separate analyses of leisure time physical activity.

**Results:**

There was no significant overall association between leisure time physical activity and risk for knee or hip replacement due to OA over the 11-year observation time. For women only, the adjusted RR (95% CI) for hip replacement was 0.66 (0.48, 0.89) (fourth vs. first quartile), indicating a lower risk of hip replacement in those with the highest compared with the lowest physical activity. The most commonly reported activities were walking, bicycling, using stairs, and gardening. Walking was associated with a lower risk of hip replacement (adjusted RR 0.76 (95% CI 0.61, 0.94), specifically for women (adjusted RR 0.75 (95% CI 0.57, 0.98)).

**Conclusions:**

In this population-based study of middle-aged men and women, leisure time physical activity showed no consistent overall relationship with incidence of severe knee or hip OA, defined as joint replacement due to OA, over 11 years. For women, higher leisure time physical activity may have a protective role for the incidence of hip replacement. Walking may have a protective role for hip replacement, specifically for women.

## Background

Osteoarthritis (OA) is common and the future prevalence is expected to increase substantially due to an aging population and an increased prevalence of obesity. It is, therefore, important to identify putative predictors in order to prevent or slow the disease process. Physical activity is a modifiable factor with beneficial effects on overall health. Despite numerous studies and several reviews [1-5], the potentially harmful or preventive effects of physical activity on development of OA are unclear. Recent systematic reviews report that some studies show an association between physical activity and an increased risk for knee or hip OA, while others show no association or that physical activity has a protective role [1-5]. Different study designs and various criteria for OA may explain these conflicting results.

Because cross-control studies are associated with limitations, such as recall bias, longitudinal prospective studies are needed to determine whether physical activity prevents or causes OA. Some studies with prospective designs have used radiographic knee OA [6], self-reported physician-diagnosed knee or hip OA [7,8], or knee or hip replacement due to OA [9-11] for case identification. Radiological findings are poorly correlated with the patient’s perceived symptoms, and the validity of self-reported OA can be questioned. Joint replacement due to OA is often considered an acceptable surrogate indicator of severe symptomatic, end-stage OA of significant economic disease burden.

Few longitudinal prospective studies have explored the effects of physical activity during leisure time on the risk of future knee or hip replacement due to OA [9-11]. These studies observed no association between leisure time physical activity and the risk of hip replacement due to OA [9-11], while there seemed to be an association with the risk of knee replacement [9].

The aim of this large population-based prospective cohort study was to explore the influence of leisure time physical activity on severe knee or hip OA, defined as knee or hip replacement due to OA.

## Methods

### Study population

The Malmö Diet and Cancer (MDC) cohort, including 28449 participants (17203 (60%) women, 11246 (40%) men) [12], constituted the study population (Table 1). The MDC is a population-based cohort established between 1991 and 1996. Characteristics of all participants (41%) and non-participants were reported [13]. The participants were representative of the eligible population regarding the prevalence of overweight, smoking, educational level, type of employment and marital status, but the mortality rate was higher among the non-participants [13]. Between 1991 and 1996, the respondents participated in a baseline examination at the screening center and filled in a self-administered questionnaire including questions on leisure time physical activity. The participants were followed until 2005 (Table 1). In the present report, participants who had been surgically treated due to hip or knee OA before the baseline examination were excluded (knee n = 129, hip n = 184).

**Table 1:** Characteristics of the study population

**Characteristic**	**Men (n = 11246)**	**Women (n = 17203)**	**All (n = 28449)**
Age at baseline, years (mean (SD), range)	59 (7.0), 45–74	57 (7.9), 44–74	58 (7.6), 44–74
BMI, kg/m^2^, at baseline (mean (SD), range)	26.3 (3.5), 13.9–50.7	25.4 (4.2), 14.0–50.9	25.8 (4.0), 13.9–50.9
Time from baseline to final follow-up, years (mean (SD))	11 (2.9)	11 (2.4)	11 (2.6)

BMI, body mass index.

The research ethics committee at Lund University approved the MDC study (LU 51–90) and the participants signed a written informed consent.

### Leisure time physical activity

At baseline, leisure time physical activity was assessed as described [14]. A questionnaire, adapted from the Minnesota Leisure Time Physical Activity Questionnaire, was used [15]. The participants were asked to fill in how many minutes per week they spent on each of 18 different activities during each of the four seasons. Each activity was multiplied by an activity-specific factor, representing assumed energy consumption (e.g., the factor for ball sports was higher than that for walking). All activities for each individual were then added together, creating an overall leisure time physical activity score. Thus, this score, represents the aggregated assumed energy consumption, taking both duration and intensity of physical activities into account [16]. The score was divided into quartiles: low (Q1), low–moderate (Q2), moderate–high (Q3), and high physical activity (Q4). The questionnaire showed acceptable validity, equal to that of a 3-point scale of physical activity (low, moderate, high), compared with accelerometry [17]. Reproducibility, assessed by telephone interview in the sample reporting the highest physical activity, was high (93%) [16].

### Definition of knee and hip osteoarthritis

Knee OA was defined as a first knee replacement or high tibial osteotomy in combination with a concomitant diagnosis of OA, and hip OA was defined as a first hip replacement in combination with a concomitant diagnosis of OA [18]. That is, the surgery indication was OA, and all other diagnoses and indications were excluded. Patients with more than one hip or knee replacement were only counted once. The exact follow-up time was calculated, separately for knee and hip OA, from the baseline examination until the first OA surgery, emigration from Sweden, death or December 31 2005, whichever came first [18]. Information on knee and hip replacement for OA and mortality were based on record linkage with the Swedish hospital discharge register and the Swedish causes of death register. Subjects who moved out of Sweden were censored at the day of emigration.

### Statistical analysis

Cox’s proportional hazards model was used to assess the incidence of total joint replacement, or osteotomy (knee), in separate analyses of leisure time physical activity. The relationships were adjusted for potential confounding factors, i.e., age, gender, BMI, education, smoking, marital status. Age and BMI were entered as continuous variables in the model. Smoking was categorized into never smokers, ex-smokers, occasional smokers, or regular smokers. Marital status was categorized into married, single, divorced or widowed. Education was categorized into less than 9 years education, 9–11 years education, and completed college or university education. All comparisons were two-sided and P-values less than or equal to 0.05 were considered statistically significant.

## Results

### Incidence of total knee replacement due to OA

**Figure 1:**
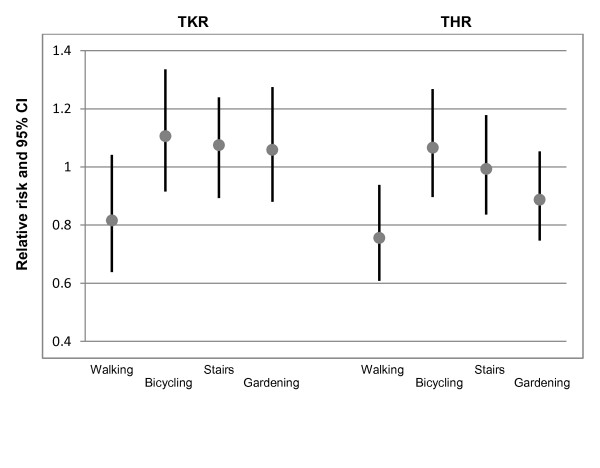
**Adjusted relative risks and 95% CI for total knee replacement (TKR) or hip replacement (THR) due to OA.** Association between the four most commonly reported activities (walking bicycling, using stairs, and gardening), respectively, and TKR/THR. Walking was associated with a lower risk of THR.

There were 27813 subjects available for the analysis of the incidence of knee OA from baseline to final follow-up, i.e., over 11 years (missing data n = 507). 467 of these were treated with knee replacement or high tibial osteotomy. There was a small, non-significant, trend of higher risk of knee OA with higher leisure time activity level, with and without adjustment for confounding factors (adjusted RR (95% CI) in the fourth quartile was 1.27 (0.97, 1.66), p trend 0.087) (Table 2). The four most commonly reported activities were walking, bicycling, using stairs, and gardening. None of these activities were associated with knee replacement or osteotomy (Figure 1).

**Table 2:** Incidence of knee replacement/high tibial osteotomy (TKR) due to osteoarthritis in relation to leisure time physical activity

**Physical activity leisure, baseline**	**Cases, n**	RR (95% CI)*	***P*, trend**	**RR (95% CI)†**	***P*, trend**	**RR (95% CI) ‡**	***P*, trend**
**TKR**							
***All***							
Low (n = 6934)	104	1.0	0.398	1.0	0.898	1.0	0.087
Low-moderate (n = 6949)	122	1.15 (0.88, 1.49)		1.13 (0.87, 1.47)		1.31 (1.00, 1.70)	
Moderate-high (n = 6973)	124	1.16 (0.89, 1.51)		1.13 (0.87, 1.46)		1.36 (1.04, 1.77)	
High (n = 6957)	117	1.13 (0.86, 1.47)		1.03 (0.79, 1.34)		1.27 (0.97, 1.66)	
***Men***							
Low (n = 2777)	34	1.0	0.046	1.0	0.390	1.0	0.163
Low-moderate (n = 2639)	36	1.09 (0.68, 1.74)		1.03 (0.64, 1.64)		1.08 (0.68, 1.73)	
Moderate-high (n = 2604)	48	1.47 (0.95, 2.29)		1.31 (0.84, 2.03)		1.44 (0.93, 2.25)	
High (n = 2925)	51	1.44 (0.94, 2.22)		1.15 (0.74, 1.77)		1.28 (0.83, 1.99)	
***Women***							
Low (n = 4157)	70	1.0	0.648	1.0	0.548	1.0	0.349
Low-moderate (n = 4310)	86	1.17 (0.85, 1.60)		1.18 (0.86, 1.62)		1.42 (1.03, 1.96)	
Moderate-high (n = 4369)	76	1.02 (0.73, 1.41)		1.03 (0.74, 1.43)		1.29 (0.93, 1.79)	
High (n = 4032)	66	0.97 (0.69, 1.36)		0.94 (0.67, 1.32)		1.22 (0.87, 1.72)	

Incidence of knee replacement, or high tibial osteotomy, due to osteoarthritis during a mean follow-up of 11 years, in relation to leisure time physical activity at baseline (low (Q1), low–moderate (Q2), moderate–high (Q3), and high physical activity (Q4)).

*crude, † adjusted for age, gender, ‡ adjusted for age, gender (for all subjects only), BMI, education, smoking, marital status.

### Incidence of hip replacement due to OA

There were 27760 subjects available for the analysis of the incidence of hip OA from baseline to final follow-up (missing data n = 505). 557 of these were treated with hip replacement. There was a small, non-significant, trend of lower risk of hip OA with higher leisure time activity level, with and without adjustment for confounding factors (Table 3). The lower risk was particularly seen in women, where the crude and adjusted RR values reached statistical significance (adjusted RR (95% CI) in the fourth quartile was 0.66 (0.48, 0.89), p trend 0.008). Walking was associated with a lower risk of hip replacement (Figure 1). This was specifically seen in women (adjusted RR 0.75 (95% CI 0.57, 0.98). The adjusted RR (95% CI) for men was 0.77 (0.54, 1.1).

**Table 3:** Incidence of hip replacement (THR) due to osteoarthritis in relation to leisure time physical activity

**Physical activity leisure, baseline**	**Cases, n**	RR (95% CI)*	***P*, trend**	**RR (95% CI)†**	***P*, trend**	**RR (95% CI) ‡**	***P*, trend**
**THR**							
***All***							
Low (n = 6935)	150	1.0	0.159	1.0	0.045	1.0	0.192
Low-moderate (n = 6933)	144	0.94 (0.75, 1.18)		0.93 (0.74, 1.17)		0.97 (0.77, 1.23)	
Moderate-high (n = 6957)	134	0.87 (0.69, 1.10)		0.85 (0.68, 1.08)		0.91 (0.72, 1.16)	
High (n = 6935)	129	0.86 (0.68, 1.09)		0.80 (0.63, 1.01)		0.86 (0.68, 1.10)	
***Men***							
Low (n = 2777)	38	1.0	0.081	1.0	0.332	1.0	0.187
Low-moderate (n = 2634)	49	1.33 (0.87, 2.03)		1.28 (0.84, 1.95)		1.31 (0.86, 2.01)	
Moderate-high (n = 2598)	43	1.18 (0.76, 1.83)		1.09 (0.71, 1.69)		1.15 (0.74, 1.79)	
High (n = 2914)	60	1.51 (1.01, 2.27)		1.31 (0.87, 1.97)		1.40 (0.93, 2.12)	
***Women***							
Low (n = 4158)	112	1.0	0.003	1.0	0.002	1.0	0.008
Low-moderate (n = 4299)	95	0.81 (0.61, 1.06)		0.82 (0.62, 1.07)		0.85 (0.64, 1.12)	
Moderate-high (n = 4359)	91	0.76 (0.58, 1.01)		0.78 (0.59, 1.02)		0.82 (0.62, 1.09)	
High (n = 4021)	69	0.63 (0.47, 0.85)		0.62 (0.46, 0.83)		0.66 (0.48, 0.89)	

Incidence of hip replacement due to osteoarthritis during a mean follow-up of 11 years, in relation to leisure time physical activity at baseline (low (Q1), low–moderate (Q2), moderate–high (Q3), and high physical activity (Q4)).

*crude, † adjusted for age, gender, ‡ adjusted for age, gender (for all subjects only), BMI, education, smoking, marital status.

## Discussion

The results from our large, prospective population-based cohort study showed a small, non-significant, trend of higher risk of knee replacement due to OA with higher leisure time physical activity, assessed in men and women at a mean age of 58 years. For hip OA, an opposite trend was found, and for women, higher leisure time physical activity was associated with a reduced risk of hip replacement. Walking was associated with a reduced risk for hip replacement, specifically for women. Our findings indicate a possible difference in effects of physical activity on the risk of knee and hip replacement due to OA. Walking, which involves low joint loading, may have a protective role for hip replacement. The possible gender difference in effects of physical activity, denote that men and women should be analyzed separately.

There was a trend of higher risk for knee replacement due to OA with higher leisure time physical activity over 11 years. However, the results were non-significant, and the risk for knee replacement increased with higher physical activity by a factor less than two (adjusted RRs between 1.08 and 1.44), indicating that leisure time physical activity was not a major risk factor for knee replacement/osteotomy. Wang et al. [9] recently reported that increasing levels of leisure time physical activity were associated with an increased risk of knee replacement due to OA over approximately 10 years. The effect seemed to be related to vigorous activity but not to less vigorous activity or walking [9]. Although their values reached statistical significance, the effect was small (HRs between 1.08 and 1.47) [9] and similar to that observed in our study. Previous case–control studies reported either an increased risk of knee replacement with high exposure to sports [19], or a reduced risk with increasing cumulative hours of recreational physical activity [20]. Longitudinal prospective studies, with similar follow-up time as in our study, reported that physical activity appeared to have little effect on OA risk in middle-aged and older subjects, measured as radiographic or symptomatic knee OA [6], or self-reported physician-diagnosed knee or hip OA [7].

For the hip, there was a small, non-significant, trend of lower risk for OA with higher leisure time physical activity over 11 years, indicating that leisure time physical activity was not a risk factor for hip replacement. This is in line with previous prospective cohort studies, reporting that leisure time physical activity was not a risk factor for hip replacement due to OA [9-11]. The values in the present study reached statistical significance for women, showing a 34% lower risk of hip OA in those with the highest compared with the lowest physical activity (lowest RR 0.66, lowest CI 0.48). This indicates a protective role of leisure time physical activity for the incidence of hip replacement in women. Further studies are needed to confirm this possible gender difference.

Walking was associated with a reduced risk for hip replacement, specifically in women. Physical activity involving higher joint loads (soccer, weight lifting), regular or intense exercise (elite, ex-athlete, physical education teachers), and frequent knee-bending activities may be associated with an increased risk for OA [1,2,21-24], although this risk is less than that for previous joint injury and overweight [2]. Physical activity involving lower joint loads, (long-distance running, swimming, walking, golf), and moderate exercise do not appear to increase the risk of knee or hip OA development or progression, or may even have a protective role [2,4,5,8,22,24,25]. Participation in sports activities, such as ball games, was not particularly common in our cohort, which is likely related to the age of the participants (mean 58 years at baseline). Therefore, comparison of activities involving high or low joint loading could not be performed.

The large size of the cohort and the prospective design are major strengths of the present study [18]. The participants were representative of the eligible population, and those who had been surgically treated due to hip or knee OA before the baseline examination were excluded. We used a case definition of knee replacement (or high tibial osteotomy) or hip replacement due to OA; a definition which is highly related to the disease burden of OA. A limitation of this definition is that only a small proportion of the total OA population undergoes knee or hip replacement (“the tip of the iceberg”). However, it has the advantage of an unambiguous relationship with the OA disease burden. So, from our results, we can draw conclusions for this group only, and not for those with possible symptomatic or radiographic OA and less severe disease, or those with severe OA that for a variety of reasons (e.g., not willing to consider TJR) have not undergone joint replacement [26]. The presence and effects of such patient selection bias cannot be ruled out, but it is difficult to value whether this would cause an over- or underestimation of our results.

The Swedish hospital discharge register was used for case-retrieval. This register was active during the entire follow-up period and covers all Swedish hospitals. A validation study reported that at least 95% of primary knee and hip joint replacements were included in the register, and that the diagnostic misclassification was about 5% for hip replacement [27]. Primary OA was the diagnosis for more than 85% and 75% of all primary knee and hip joint replacements, respectively, in Sweden during the follow-up period [18]. We therefore believe that the bias in our study due to misclassification of OA is small, likely not influencing the results.

The progression from symptomatic OA to a joint replacement occurs over approximately a decade [28], suggesting sufficient follow-up time in our study. However, some persons entering the present study may have had early OA symptoms, possibly limiting their physical activity. This may have caused an underestimation of our results.

Obesity is a risk factor for OA of the knee or hip. In the same cohort as that used in the present study, the risk for knee or hip OA was 4–7 and 2–3 times higher, respectively, in people with obesity compared to those with normal weight [18]. BMI was well documented, and adjusted for in the present study.

There are a number of potential limitations to our study. History of joint trauma was not recorded at the time of the baseline examination. Previous knee injury is a well known risk factor for knee OA [29], and when adjusted for, regular physical activity may protect against knee OA [30]. Also for hip OA, a history of hip joint trauma is considered a risk factor [2,24]. Because hip injuries are less common than knee injuries, the impact of previous hip injury is likely small in the current study. However, we cannot rule out that previous joint injury, particularly to the knee, may have affected our results. This may constitute one explanation for the opposing trends on the effects of physical activity and the risk of future knee and hip replacement. This is in line with a previous prospective cohort study, reporting that higher levels of physical activity were associated with an increased risk of knee replacement due to OA, whereas no such association was found for the hip [9]. The different anatomical characteristics and function of the hip and knee joints may comprise another reason for the possible different effects of physical activity on knee and hip replacement.

Another weakness of our study, and of previous studies [9-11], is that sports participation earlier in life was not adjusted for. High exposure to sports earlier in life was related to an increased risk for knee or hip OA, defined as joint replacement [19,21] radiographic OA [31,32], or medically-diagnosed self-reported hip OA [24], implying that lifetime physical activity should be adjusted for.

The influence of occupational physical activity on knee or hip OA is more consistent than that for leisure time physical activity. Systematic reviews conclude some evidence of an association between high work load and knee or hip OA [1,2,33,34]. Since we do not have data for the participants’ previous occupational physical activity, we could not control for this in the analysis. Because an urban population was included, there were likely few participants with high risk occupations, such as farming [35,36]. The interplay between physical activity at work and during leisure time and the association with knee and hip OA may be important to consider in future studies. Men with high exposure to both sports activities and occupational/leisure time physical work load may have an increased risk of knee replacement due to OA [19]. It was also reported that men whose jobs included both knee bending and higher physical demands had an increased risk of radiographic incident OA [37].

Self-report surveys to measure physical activity are associated with some limitations, such as, over-reporting, reduced accuracy for moderate physical activity, and moderate reproducibility [3]. Accelerometry provides an objective and reliable measure of the frequency, duration and intensity of physical activity, but is impractical to use in large cohorts [3]. Although validity and reliability were reported for the questionnaire used [16,17], these studies were small and further validation may be required.

Studies of this cohort [14] and other cohorts [38-40] report that higher physical activity, assessed by the questionnaire that we used, or a 3–point scale, was associated with a lower risk of cardiovascular diseases, implying that these questionnaires are sensitive in detecting important health risks. These questionnaires, and other questionnaires that are used in patients with hip or knee OA [41], measure the aerobic aspect of physical activity, which is relevant for cardiovascular diseases but may not be appropriate for OA. Because current instruments may not have adequate measurement properties for measuring physical activity as risk factor for OA development [41], this may be a reason for failing to find a consistent association with knee or hip OA in our study and in previous prospective cohort studies [6,7,9-11]. Questionnaires accurately assessing physical activity in terms of type, frequency, intensity, and joint load seem to be relevant and important for studying the association with OA. Such questionnaires may also determine whether the association of physical activity with OA is linear or not, as it may be assumed that both very high and very low joint loads are risk factors for OA.

## Conclusions

In our longitudinal prospective population-based cohort study of middle-aged men and women, leisure time physical activity showed no consistent overall relationship with incidence of severe knee or hip OA, defined as joint replacement due to OA, over 11 years. For women, higher leisure time physical activity, and particularly walking, may have a protective role for the incidence of hip replacement.

## Competing interests

Declared. GE, MG, and JR are employees of AstraZeneca. EA, EMR, LSL have no conflicts of interest to report.

## Authors’ contributions

All authors contributed to the conception and design of the study. EA performed the data analysis and drafted the manuscript. All authors contributed to interpretation of the data, contributed to critical revision of the article for important intellectual content, and read and approved the final version.

## Pre-publication history

The pre-publication history for this paper can be accessed here:

http://www.biomedcentral.com/1471-2474/13/73/prepub
